# The rise of robotics: Surgical approaches for rectal cancer over time

**DOI:** 10.1007/s00464-025-12065-w

**Published:** 2025-08-25

**Authors:** Emily F. Simon, Kristen M. Westfall, Kamil Erozkan, Lauren Henke, Meagan Costedio, Trevor Teetor, Jennifer Eva Selfridge, Emily Steinhagen, Ronald Charles

**Affiliations:** 1 Division of Colorectal Surgery, Department of Surgery, University Hospitals Cleveland Medical Center, 11100 Euclid Ave, Cleveland, OH 44106 USA; 2 Department of Radiation Oncology, Seidman Cancer Center, University Hospitals Cleveland Medical Center, Cleveland, OH USA; 3 Department of Hematology and Oncology, Seidman Cancer Center, University Hospitals Cleveland Medical Center, Cleveland, OH USA

**Keywords:** Robotic surgery, Locally advanced rectal cancer, Oncologic resection, Minimally invasive surgery, Surgical trends, Oncologic outcomes

## Abstract

**Background:**

Robotic surgery for rectal cancer has gained attention for addressing anatomic challenges, though concerns about cost, operative time, and oncologic outcomes persist. Studies on laparoscopic surgery initially showed mixed results regarding long-term oncologic safety, but current data is favorable. Evidence supporting the robotic approach remains limited. This study examined trends in robotic surgery, including facilitation of high-quality oncologic resection, defined by lymph node yield and negative margins, using National Cancer Database (NCDB) data.

**Methods:**

A multicenter cohort study analyzed 87,611 patients with stage I–III rectal cancer undergoing definitive resection between 2010 and 2021. Outcomes included robotic surgery rates, high-quality oncologic resection (defined using lymph node yield and surgical margins), length of stay, 30-day readmission, and mortality. Outcomes were stratified into tertiles (2010–2013, 2014–2017, 2018–2021) and analyzed via univariate analysis and multivariate logistic regressions.

**Results:**

Robotic surgery use rose from 10.1% (2010–2013) of oncologic resection to 45.7% (2018–2021) (*p* < 0.001). High-quality oncologic resection improved over time: 65.4% to 75.3% (*p* < 0.001). Compared to open surgery, robotic surgery had 1.39 times higher odds of achieving high-quality oncologic resection (*p* < 0.001). Despite both improving over time, robotics had higher odds than laparoscopic surgery of high-quality oncologic resection in each tertile (e.g., 2018–2021 OR 1.64, CI 1.49–1.81 vs. OR 1.44, CI 1.30–1.61; respectively). High-volume centers further increased the likelihood of successful outcomes (OR 1.6, *p* < 0.001). Robotic and laparoscopic approaches were associated with significantly lower mortality compared to open surgery (OR 0.63, *p* < 0.001; OR 0.72, *p* < 0.001, respectively).

**Conclusion:**

Robotic surgery has significantly improved in oncologic measures, including rates of quality surgical resection and patients’ overall survival, over the past decade, outperforming laparoscopic and open techniques. These findings underscore the growing role of robotic surgery in rectal cancer care and the need for formalized training and credentialing to ensure continued progress and equitable access to this technology.

**Graphical Abstract:**

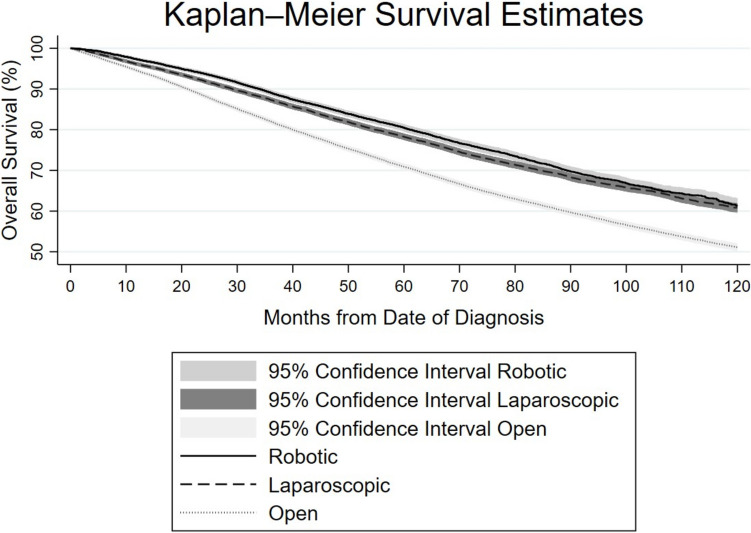

The increasing use of robotic surgery in rectal cancer care has prompted ongoing evaluation regarding its oncologic efficacy and clinical value. As with any emerging surgical modality, robotic-assisted techniques are subject to scrutiny—though perhaps not to the same extent as when laparoscopic surgery first diverged from open approaches [1, 2]. Unlike the paradigm shift from open to laparoscopic surgery, where fundamental changes in operative access and technique occurred, the transition from laparoscopy to robotics is more nuanced, building on the same minimally invasive principles with the potential for improved ergonomics, visualization, and dexterity. Nevertheless, given the well-established safety and efficacy of both open and laparoscopic techniques for rectal cancer, it remains essential to rigorously assess whether robotic surgery offers meaningful advantages that justify its broader adoption, especially in light of its cost and resource demands. While the technical evolution may be more incremental, confirming the oncologic soundness and clinical benefit of robotic surgery remains a critical step in the natural progression of surgical innovation.

Robotic surgery presents a compelling option for the oncologic resection of rectal cancer, particularly given the technical challenges inherent to pelvic surgery. For example, robotic-assisted procedures for prostate cancer, another pelvic disease, have demonstrated advantages in outcomes for patients, including less blood loss, urinary incontinence and erectile dysfunction [3, 4]. Additionally, when optimized, the superior ergonomics of robotic systems can alleviate surgeon fatigue and physical distress during pelvic operations compared to open or laparoscopic approaches [5–7]. However, robotic surgery is frequently criticized for its high costs, long operative times, and the ongoing physical demands on surgeons, leading some to question whether its technical advantages translate into meaningful clinical benefits [5, 7–11]. While these features offer a promising platform for complex oncologic resections, robotic surgery must demonstrate clear oncologic superiority—both in achieving optimal resection outcomes and minimizing postoperative complications—to justify its widespread adoption and associated resource allocation.

The evidence supporting robotic surgery in rectal cancer remains mixed and continues to evolve, mirroring the early trajectory of laparoscopic surgery [12–17]. To date, the most robust data comes from a single large randomized controlled trial, which demonstrated no difference in conversion to open surgery between laparoscopic and robotic approaches and reported comparable rates of lymph node yield, positive circumferential radial margins, and mesorectal completeness [10]. Other studies, such as a cross-sectional analysis of approximately 6000 patients, have shown lower conversion rates to open surgery with robotic techniques, but did not address oncologic or survival outcomes [18]. A meta-analysis of 20 studies corroborated the lower conversion rates observed in robotic surgery and noted reductions in hospital length of stay, time to bowel function recovery, and postoperative complications [19]. However, it also highlighted that robotic surgery had longer operative times and no significant difference in lymph node yield or circumferential margins compared to laparoscopic surgery [19]. While additional studies have affirmed the safety and feasibility of robotic surgery for rectal cancer, [6–8]there remains a lack of evidence to definitively demonstrate superior oncologic outcomes compared to laparoscopic or open approaches, leaving its broader justification unresolved [20–22].

Building on the evolving narrative of laparoscopic surgery and the current mixed evidence surrounding robotic surgery, the advantages and disadvantages of robotic approaches underscore the need for rigorous evaluation to determine its oncologic value. We hypothesized that oncologic outcomes using robotic surgery have improved over time, as the robot has gained availability and familiarity among providers. A composite variable was devised to represent high-quality oncologic resection composed of adequate lymph node yield and negative surgical margins, as each have important prognostic implications [23–28]. Using data available over the last decade from the National Cancer Database (NCDB), the current study aims to describe the rates of robotic surgery and achievement of superior oncologic resection in rectal cancer patients relative to traditional laparoscopic and open approaches.

## Materials and methods

### Data source

This was a retrospective cohort study utilizing data from the National Cancer Database (NCDB). The NCDB is a clinical oncology database that captures roughly 72% of newly diagnosed cancers annually from over 1500 Commission-accredited cancer programs [29]. Data from 2010 to 2021 was analyzed as surgical approach (laparoscopic, robotic, open) was first collected in 2010.

### Patient population

Patients with Stage I through Stage III rectal adenocarcinoma who underwent surgical resection were included. Patients with other tumor histology (ICD codes other than 8140–8147, 8210–8213, or 8220–8221), metastatic disease or unknown metastatic (M) stage were excluded. All patients were age 18 or older, and patients age 90 or older were coded as 90 years of age as predefined in the NCDB. Other demographic variables included sex (defined as binary male/female), race (defined as non-Hispanic White, non-Hispanic Black, Hispanic and other), Comorbidity Score (defined as an ordinal variable ranging from 0 to 3 or more, with 0 representing no comorbidities), patient residence (metropolitan, urban, rural or unknown), insurance status (uninsured, private, Medicaid/Medicare, other government, or unknown) and income (defined as median income in quartiles). Overall clinical stage (cTNM) and clinical nodal (cN) stage were also defined. Patients were divided into three categories based on year of diagnosis: 2010 to 2013, 2014 to 2017 and 2018 to 2021 to allow for comparisons of surgical outcomes over time.

### Treatment and surgery

Characteristics of the facility where a patient received care were defined as facility type (community cancer program, comprehensive community cancer program, academic or research program, integrated network cancer program or unknown) and facility surgical volume (defined by dividing the patients into tertiles of low, medium or high volume of rectal oncologic resections).

Whether or not a patient received chemotherapy and/or radiation, the dosage of radiation (simplified into long course, short course or other), and the order of starting chemotherapy and/or radiation relative to surgery were defined and compared across year of diagnosis. Furthermore, treatment sequence was compared across cTNM stages I–III.

The variable “RX_HOSP_SURG_PRIM_SITE” was utilized to define surgical events. This variable captures the most definitive oncologic resection performed at the specific facility reporting a patient’s data to the NCDB. Patients were excluded if they were coded as having no surgery, local destruction or excision only, surgical type was not well defined (total proctocolectomy, NOS; proctectomy, NOS; surgery, NOS), or if it was unknown if surgery was performed or no data was provided. Remaining surgical procedures included “partial proctectomy” (low anterior resection and anterior resection), “coloanal anastomosis”, “total proctectomy” (abdominoperineal resection), and “proctectomy or proctocolectomy with resection in continuity with other organs; pelvic exenteration”. Surgical approach was defined using the variable “RX_HOSP_SURG_APPR_2010”. Categories were created using intended (i.e. planned) surgical approach: robotic, laparoscopic, or open. When appropriate, groups were further divided to separate cases completed robotically or laparoscopically from those converted to open.

### Outcomes

There were two primary outcomes. The first was changes in the proportion of robotic surgery over time. The second was a binary composite variable, defined as having both 12 or more lymph nodes removed (using the variable “REGIONAL_NODES_EXAMINED”) and negative surgical margins (using the variable “RX_SUMM_SURGICAL_MARGINS”) at the time of oncologic resection. Patients were excluded if either variable defining the composite variable was unknown or blank. Secondary outcomes included readmission within 30 days, length of hospital stay, 30-day mortality and 90-day mortality.

### Statistical analysis

All analysis was performed using StataSE v17.0 (Statacorp LLC, College Station, TX). Univariate analysis compared demographics and surgical outcomes across the three groups of patients. Pearson’s chi-square test was used to compare categorical variables while Kruskal–Wallis test was used for continuous variables.

Multivariable logistic regression models were used to determine factors associated with achieving the composite outcome, as well as readmission in 30 days, length of stay greater than 1 week, and mortality. Year of diagnosis (as a categorical variable defined above), intended surgical approach (open, laparoscopic, robotic), surgical procedure, cTNM stage, facility volume and selected demographic factors (Charlson-Deyo Comorbidity Score, race, sex, age) were used as independent predictors. To explore whether the effect of surgical approach on achieving the composite variable differed by year of diagnosis, interaction terms between surgical approach and year of diagnosis were included in the multivariable logistic regression model.

Additional regression modeling was performed utilizing the broader categorization of surgical approach (open, laparoscopic, robotic, laparoscopic converted to open, robotic converted to open). Furthermore, to better delineate the association between the composite outcome and minimal invasive approaches, another iteration of the model was conducted that utilized only robotic and laparoscopic cases (but including those converted to open), excluding cases that were performed open primarily. All regression models otherwise used the same interaction terms and independent predictors as noted above.

A Cox proportional hazards regression model was employed to analyze the relationship between surgical approach and mortality, accounting for potential confounders (patient age, year of diagnosis) and time-to-event data. Sub-analyses were performed utilizing 1) only robotic and laparoscopic cases and 2) only patients of a given cTNM stage (I, II or III).

## Results

### Demographics

**Fig. 1:**
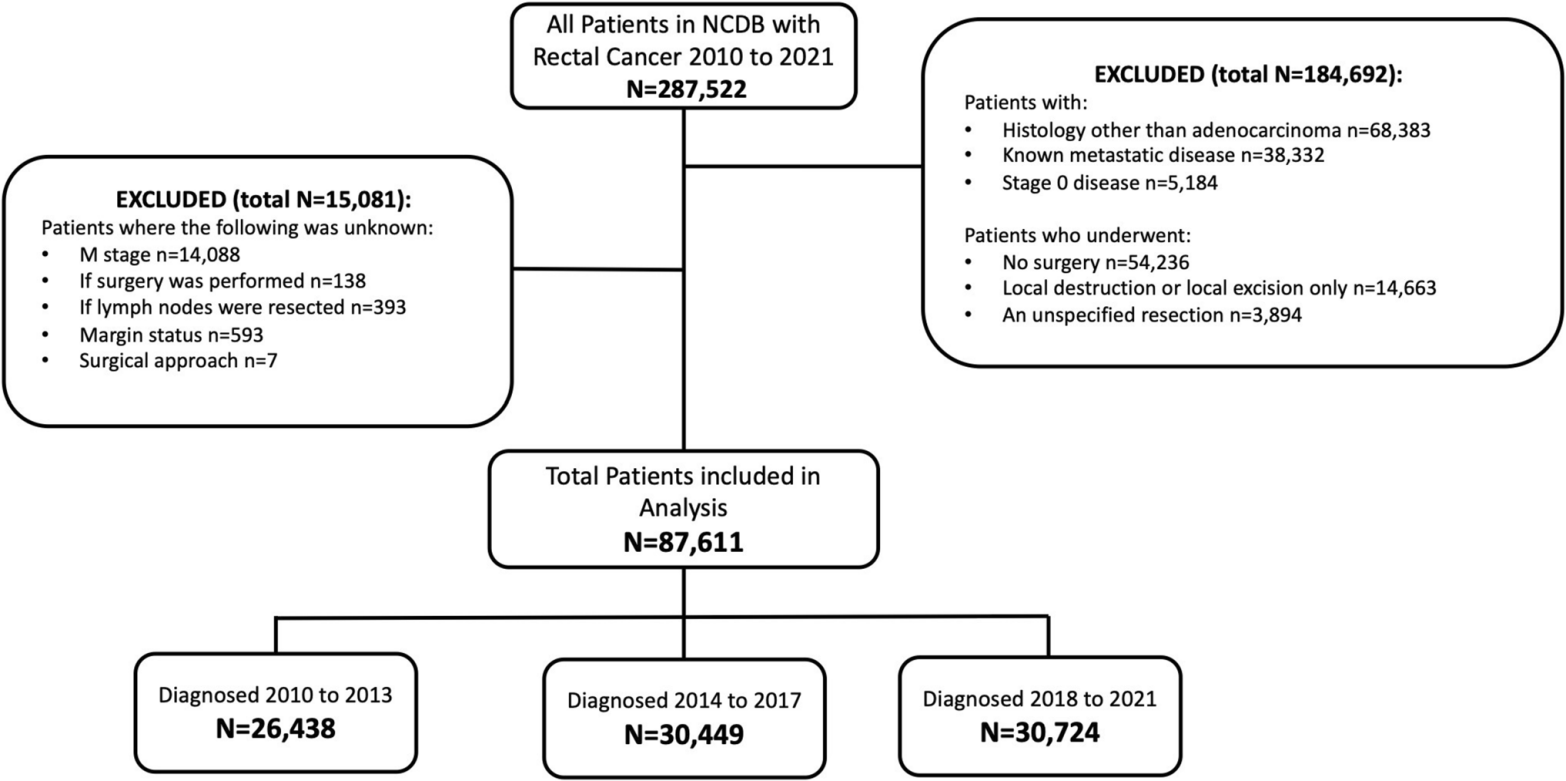
CONSORT diagram. Flowchart of patient exclusion from National Cancer Database based on study criteria; *M stage* metastatic stage

A total of 287,522 patients diagnosed with rectal cancer between 2010 and 2021 were available in the NCDB. After exclusion, 87,611 patients were included for the final analysis (Fig. 1): 26,438 diagnosed between 2010 and 2013, 30,449 diagnosed between 2014 and 2017 and 30,724 diagnosed between 2018 and 2021. Roughly 61% of patients were male, 77% were non-Hispanic White and the median patient age was 62 years old. Most patients had low Charlson-Deyo Comorbidity (CDC) scores, with greater than 92% scoring 1 or less. Most patients had clinical stage III disease (38.44%; relative to stage I (16.15%), stage II (25.21%) and unknown stage (20.19%)). Additional demographic factors and univariate analysis across patient diagnostic year groups is reported in Table 1.

**Table 1:** Univariate comparison of demographic factors across patients grouped by year of diagnosis

		2010–2013	2014–2017	2018–2021	*p*-value
		Total *N* = 26,438 *N* (% or IQR)	Total *N* = 30,449 *N* (% or IQR)	Total *N* = 30,724 *N* (% or IQR)	
Sex	Male	15,989 (60.5%)	18,720 (61.5%)	18,680 (60.8%)	0.042
	Female	10,449 (39.5%)	11,729 (38.5%)	12,044 (39.2%)	
Race	Non-Hispanic White	20,724 (78.4%)	23,689 (77.8%)	23,232 (75.6%)	< 0.001
	Non-Hispanic Black	2066 (7.8%)	2340 (7.7%)	2370 (7.7%)	
	Hispanic	1486 (5.6%)	2091 (6.9%)	2626 (8.5%)	
	Other	2162 (8.2%)	2329 (7.6%)	2496 (8.1%)	
Age at diagnosis		62 (53–72)	61 (53–70)	61 (52–70)	< 0.001
Charlson-Deyo comorbidity score	0	19,539 (73.9%)	23,045 (75.7%)	23,090 (75.2%)	< 0.001
	1	5187 (19.6%)	5204 (17.1%)	4907 (16.0%)	
	2	1228 (4.6%)	1374 (4.5%)	1497 (4.9%)	
	3 or more	484 (1.8%)	826 (2.7%)	1230 (4.0%)	
Clinical N stage	N stage 0	15,884 (60.1%)	16,952 (55.7%)	15,056 (49.0%)	< 0.001
	N stage 1	7129 (27.0%)	9077 (29.8%)	9406 (30.6%)	
	N stage 2	1379 (5.2%)	2995 (9.8%)	4883 (15.9%)	
	N stage unknown	2046 (7.7%)	1425 (4.7%)	1379 (4.5%)	
Clinical TNM stage	Stage 1	4802 (18.2%)	4621 (15.2%)	4723 (15.4%)	< 0.001
	Stage 2	7618 (28.8%)	7785 (25.6%)	6688 (21.8%)	
	Stage 3	8182 (30.9%)	11,584 (38.0%)	13,915 (45.3%)	
	Stage unknown	5836 (22.1%)	6,459 (21.2%)	5398 (17.6%)	
Patient residency	Metropolitan	20,757 (78.5%)	24,100 (79.1%)	24,445 (79.6%)	< 0.001
	Urban	4009 (15.2%)	4665 (15.3%)	4714 (15.3%)	
	Rural	574 (2.2%)	589 (1.9%)	611 (2.0%)	
	Unknown	1098 (4.2%)	1095 (3.6%)	954 (3.1%)	
Insurance	Uninsured	1192 (4.5%)	846 (2.8%)	885 (2.9%)	< 0.001
	Private	12,071 (45.7%)	14,512 (47.7%)	14,622 (47.6%)	
	Medicaid or medicare	12,583 (47.6%)	14,342 (47.1%)	14,539 (47.3%)	
	Other government	295 (1.1%)	379 (1.2%)	429 (1.4%)	
	Unknown	297 (1.1%)	370 (1.2%)	249 (0.8%)	
Income	< $46,277	4142 (15.7%)	4392 (14.4%)	4,381 (14.3%)	< 0.001
	$46,277–$57,856	5336 (20.2%)	5901 (19.4%)	5676 (18.5%)	
	$57,857–$74,062	5587 (21.1%)	6254 (20.5%)	6156 (20.0%)	
	$74,063 +	8078 (30.6%)	9254 (30.4%)	9444 (30.7%)	
	Unknown	3295 (12.5%)	4648 (15.3%)	5067 (16.5%)	

Categorical variables compared using chi−square; number of patients and percentage of total patients reported per category. Continuous variables compared using Kruskal–Wallis; median and interquartile range reported

### Treatment and surgical factors

Most patients received care at a Comprehensive Community Cancer Program (36.71%) followed by Academic or Research Centers (34.56%). Most patients received both chemotherapy (72.7%, 75.2%, 75.5%, respectively; *p* < 0.001) and radiation (67.8%, 68.0%, 68.9%, respectively; *p* < 0.001), predominately in the neoadjuvant setting (55.1%, 57.7%, 59.2%, respectively; *p* < 0.001) (Table 2). Among the patients with clinical TNM stage available, 61.9% of stage I patients had no chemotherapy or radiation, whereas 73.2% of stage II and 83.0% of stage III patients had neoadjuvant chemotherapy and radiation (plus or minus various adjuvant therapy).

**Table 2:** Univariate comparison of treatment factors across patients grouped by year of diagnosis

		Years 2010–2013	Years 2014–2017	Years 2018–2021	*p*-value
		Total *N* = 26,438 n (%)	Total *N* = 30,449 n (%)	Total *N* = 30,724 n (%)	
Facility type	Community cancer program	1417 (5.4%)	1,466 (4.8%)	1,192 (3.9%)	< 0.001
	Comprehensive community cancer program	10,097 (38.2%)	11,091 (36.4%)	10,978 (35.7%)	
	Academic/research program	8378 (31.7%)	10,672 (35.0%)	11,231 (36.6%)	
	Integrated network cancer program	5643 (21.3%)	6,071 (19.9%)	6,002 (19.5%)	
	Unknown	903 (3.4%)	1,149 (3.8%)	1,321 (4.3%)	
Surgical procedure	Partial proctectomy, including LAR	18,034 (68.2%)	21,137 (69.4%)	22,364 (72.8%)	< 0.001
	Coloanal anastomosis, sphincter preserving	2006 (7.6%)	2,182 (7.2%)	1,747 (5.7%)	
	Total Proctectomy, including APR	5756 (21.8%)	6,287 (20.6%)	5,788 (18.8%)	
	Proctectomy/proctocolectomy w/ other organs	642 (2.4%)	843 (2.8%)	825 (2.7%)	
Intended operative approach	Robotic	2680 (10.1%)	8,225 (27.0%)	14,045 (45.7%)	< 0.001
	Laparoscopic	8192 (31.0%)	10,636 (34.9%)	8,883 (28.9%)	
	Open	15,566 (58.9%)	11,588 (38.1%)	7,796 (25.4%)	
Cases converted to open*	Robotic	221 (8.2%)	498 (6.1%)	665 (4.7%)	< 0.001
	Laparoscopic	1323 (16.1%)	1,481 (13.9%)	1,082 (12.2%)	< 0.001
Hospital operative volume	Low volume	9624 (36.4%)	10,152 (33.3%)	9,835 (32.0%)	< 0.001
	Medium volume	8421 (31.9%)	10,000 (32.8%)	10,573 (34.4%)	
	High volume	8393 (31.7%)	10,297 (33.8%)	10,316 (33.6%)	
Chemotherapy	No chemotherapy	6933 (26.2%)	7,258 (23.8%)	7,293 (23.7%)	< 0.001
	Received chemotherapy	19,222 (72.7%)	22,890 (75.2%)	23,205 (75.5%)	
	Unknown	283 (1.1%)	301 (1.0%)	226 (0.7%)	
Radiation	No radiation	7850 (29.7%)	8,880 (29.2%)	9,385 (30.5%)	< 0.001
	Received radiation	17,914 (67.8%)	20,692 (68.0%)	21,171 (68.9%)	
	Unknown	674 (2.5%)	877 (2.9%)	168 (0.5%)	
Radiation course	Long course	14,284 (54.0%)	16,857 (55.4%)	16,375 (53.3%)	< 0.001
	Short course	284 (1.1%)	477 (1.6%)	1,667 (5.4%)	
	Other or unknown	11,870 (44.9%)	13,115 (43.1%)	12,682 (41.3%)	
Treatment sequence	Neoadjuvant chemo + radiation	14,568 (55.1%)	17,561 (57.7%)	18,193 (59.2%)	< 0.001
	Without any adjuvant treatment	9771 (37.0%)	11,047 (36.3%)	12,884 (41.9%)	
	With adjuvant chemo	4734 (17.9%)	6434 (21.1%)	5228 (17.0%)	
	With adjuvant chemo + radiation	63 (0.2%)	80 (0.3%)	81 (0.3%)	
	No chemotherapy or radiation	6356 (24.0%)	6,748 (22.2%)	6782 (22.1%)	
	Adjuvant chemo + radiation, no neoadjuvant treatment	1995 (7.5%)	1585 (5.2%)	1229 (4.0%)	
	Adjuvant chemo only	1103 (4.2%)	1429 (4.7%)	1667 (5.4%)	
	Neoadjuvant chemo only	261 (1.0%)	471 (1.5%)	713 (2.3%)	
	Neoadjuvant + adjuvant chemo only	70 (0.3%)	190 (0.6%)	183 (0.6%)	
	Other	749 (2.8%)	976 (3.2%)	1,188 (3.9%)	
	Unknown order in part or full/missing data	1336 (5.1%)	1489 (4.9%)	769 (2.5%)	
Optimal oncologic resection	Failed	9160 (34.6%)	8460 (27.8%)	7597 (24.7%)	< 0.001
	Achieved	17,278 (65.4%)	21,989 (72.2%)	23,127 (75.3%)	

Categorical variables compared using chi-square; number of patients and percentage of total patients reported per category; *percentages represent percent of total intended robotic or laparoscopic procedures, respectively and corresponding p-values represent the chi-square analysis of cases not converted to open compared to those converted to open across the time categories for robotic or laparoscopic cases, respectively. *Chemo* chemotherapy

Over the study period, 39.89% of patients underwent open surgery, 27.19% underwent laparoscopic surgery, 26.90% underwent robotic surgery and 6.02% received open surgery after conversion (1.58% robotic to open, 4.44% laparoscopic to open). Most patients underwent a partial proctectomy (low anterior resection or anterior resection), with 68.2% in 2010–2013, 69.4% in 2014–2017 and 72.8% in 2018–2021 (*p* < 0.001). Additional univariate analysis across patient diagnostic year groups is reported in Table 2.

### Robotic surgery over time and achieving the composite outcome

The proportion of patients undergoing robotic surgery increased significantly overtime, from 10.1% in 2010–2013 and 27.0% in 2014–2017 to 45.7% in 2018–2021 (*p* < 0.001), with the proportion requiring conversion to open surgery decreasing over time (8.2%, 6.1%, 4.7%, respectively, *p* < 0.001) (Table 2). The proportion of patients who underwent surgery that achieved the composite outcome of high-quality oncologic resection also increased across time periods (65.4%, 72.2%, 75.3%, *p* < 0.001) (Table 2), including amongst the group of patients who underwent any robotic surgery (i.e. robotic or robotic converted to open; 68.8%, 76.3%, 78.5%, respectively; *p* < 0.001).

On multivariable logistic regression modeling, patients diagnosed in 2014–2017 and 2018–2021 had higher odds of receiving surgery that achieved the composite outcome relative to 2010–2013 (OR 1.28, CI 1.23–1.33; OR 1.43, CI 1.37–1.49, respectively). Relative to planned open surgery, intended robotic approach had the greatest odds of achieving the composite outcome, with an increase over time observed when including an interaction term between intended surgical approach and year of diagnosis (2010–2013 OR 1.17, CI 1.06–1.29 compared to 2018–2021 OR 1.92, CI 1.81–2.04). The intended laparoscopic approach also had increasing odds of achieving the composite over time relative to open surgery (2010–2013 OR 1.06, CI 0.99–1.13 compared to 2018–2021 OR 1.70, CI 1.59–1.82). Patients who received resections at high volume centers had 1.62 odds (CI 1.55–1.69) of receiving the composite outcome relative to the bottom tertile (Table 3).

**Table 3:** Multivariable logistic regression model predicting achievement of the composite outcome following oncologic resection

		Odds ratio	*p*-value	Confidence interval	
Intended operative approach by year of diagnosis					
2010–2013	Open	reference			
	Robotic	1.17	0.00	1.06	1.29
	Laparoscopic	1.06	0.09	0.99	1.13
2014–2017	Open	1.21	0.00	1.14	1.28
	Robotic	1.69	0.00	1.58	1.81
	Laparoscopic	1.43	0.00	1.35	1.53
2018–2021	Open	1.23	0.00	1.15	1.31
	Robotic	1.92	0.00	1.81	2.04
	Laparoscopic	1.70	0.00	1.59	1.82
Surgical procedure by stage					
Partial proctectomy (LAR or AR)	Stage 1	reference			
	Stage 2	0.83	0.00	0.78	0.87
	Stage 3	0.96	0.14	0.91	1.01
Coloanal anastomosis	Stage 1	1.52	0.00	1.28	1.80
	Stage 2	0.85	0.01	0.75	0.95
	Stage 3	1.01	0.89	0.91	1.11
Total proctectomy (apr)	Stage 1	1.11	0.07	0.99	1.23
	Stage 2	0.64	0.00	0.59	0.68
	Stage 3	0.74	0.00	0.69	0.79
Pelvic exenteration	Stage 1	1.43	0.08	0.96	2.15
	Stage 2	0.51	0.00	0.44	0.60
	Stage 3	0.72	0.00	0.63	0.82
Hospital operative volume					
	Low volume	reference			
	Medium volume	1.28	0.00	1.23	1.33
	High volume	1.62	0.00	1.56	1.69
Charlson-Deyo comorbidity score					
	0	reference			
	1	0.99	0.58	0.94	1.03
	2	0.96	0.27	0.88	1.04
	3 or more	0.99	0.90	0.90	1.10
Race					
	Non-Hispanic White	reference			
	Non-Hispanic Black	0.93	0.02	0.87	0.99
	Hispanic	1.02	0.56	0.95	1.09
	Other	1.02	0.46	0.96	1.09
Sex					
	Male	reference			
	Female	1.05	0.00	1.02	1.09
Age					
	Age < 56	reference			
	Age 56 to 66	0.81	0.00	0.77	0.84
	Age 67 +	0.75	0.00	0.72	0.78

*LAR* low anterior resection, *AR* anterior resection, *APR* abdominoperineal resection

Utilizing only patients who had minimally invasive surgery, patients who underwent laparoscopic surgery had lower odds of receiving the composite outcome (OR 0.87, CI 0.83–0.91) relative to robotic surgery. When including an interaction term between intended surgical approach and year of diagnosis, both robotic and laparoscopic surgery had improved odds of receiving the composite variable over time, relative to robotic surgery in 2010–2013. However, robotic surgery had better odds than laparoscopic surgery at all time points (2014–2017 OR 1.45, CI 1.30–1.61 vs. OR 1.22, CI 1.10–1.35; 2018–2021 OR 1.64, CI 1.49–1.81 vs. OR 1.44, CI 1.30–1.61; respectively) (Table 4).

**Table 4:** Multivariable logistic regression model predicting achievement of the composite outcome following oncologic resection in minimally invasive surgeries

		Odds ratio	*p*-value	Confidence interval	
Intended operative approach by year of diagnosis					
2010–2013	Robotic	reference			
	Laparoscopic	0.89	0.04	0.81	0.99
2014–2017	Robotic	1.45	0.00	1.30	1.61
	Laparoscopic	1.22	0.00	1.10	1.35
2018–2021	Robotic	1.64	0.00	1.49	1.81
	Laparoscopic	1.44	0.00	1.30	1.61
Surgical procedure by stage					
Partial proctectomy (LAR or AR)	Stage 1	reference			
	Stage 2	0.74	0.00	0.69	0.80
	Stage 3	0.88	0.00	0.82	0.94
Coloanal anastomosis	Stage 1	1.43	0.00	1.15	1.77
	Stage 2	0.80	0.01	0.69	0.94
	Stage 3	0.91	0.13	0.80	1.03
Total proctectomy (APR)	Stage 1	1.02	0.76	0.88	1.19
	Stage 2	0.56	0.00	0.51	0.61
	Stage 3	0.66	0.00	0.61	0.72
Pelvic exenteration	Stage 1	1.47	0.25	0.76	2.85
	Stage 2	0.40	0.00	0.30	0.53
	Stage 3	0.74	0.01	0.59	0.92
Hospital operative volume					
	Low volume	reference			
	Medium volume	1.22	0.00	1.16	1.29
	High volume	1.50	0.00	1.42	1.59
Charlson-Deyo comorbidity score					
	0	reference			
	1	0.97	0.29	0.91	1.03
	2	0.93	0.18	0.84	1.03
	3 or more	0.97	0.72	0.85	1.12
Race					
	Non-Hispanic White	reference			
	Non-Hispanic Black	0.89	0.01	0.82	0.97
	Hispanic	0.96	0.37	0.88	1.05
	Other	1.02	0.66	0.94	1.10
Sex					
	Male	reference			
	Female	1.08	0.00	1.03	1.13
Age					
	Age < 56	reference			
	Age 56 to 66	0.80	0.00	0.76	0.85
	Age 67 +	0.74	0.00	0.70	0.79

*LAR* low anterior resection, *AR* anterior resection, *APR* abdominoperineal resection

### 30-day readmission and length of stay

Patients whose surgery was converted to open had higher odds of 30-day readmission compared to planned open surgery (robotic converted to open OR 1.57, CI 1.28–1.92; laparoscopic converted to open OR 1.29, CI 1.12–1.48), whereas odds of readmission were similar in robotic (OR 1.08, CI 1.00–1.17) and laparoscopic (OR 0.98, CI 0.90–1.06) approach compared to planned open surgery (Table 5).

**Table 5:** Multivariable logistic regression model predicting 30-day readmission

	Odds ratio	*p*-value	Confidence interval	
*Year of diagnosis*				
Years 2010–2013	reference			
Years 2014–2017	0.91	0.02	0.85	0.99
Years 2018–2021	0.79	0.00	0.73	0.86
*Operative approach*				
Open or Unknown approach	reference			
Robotic	1.08	0.05	1.00	1.17
Robotic, converted to open	1.57	0.00	1.28	1.92
Laparoscopic	0.98	0.58	0.90	1.06
Laparoscopic, converted to open	1.29	0.00	1.12	1.48
*Surgical Procedure*				
Partial proctectomy (LAR or AR)	reference			
Coloanal anastomosis	1.34	0.00	1.20	1.49
Total proctectomy (APR)	1.13	0.00	1.05	1.21
Pelvic exenteration	1.39	0.00	1.18	1.64
*Clinical Stage*				
Stage 1	reference			
Stage 2	1.18	0.00	1.08	1.28
Stage 3	1.10	0.02	1.01	1.20
*Hospital Operative Volume*				
Low volume	reference			
Medium volume	1.15	0.00	1.07	1.24
High volume	1.03	0.45	0.95	1.11
*Charlson-Deyo Comorbidity Score*				
0	reference			
1	1.37	0.00	1.27	1.47
2	1.47	0.00	1.28	1.67
3 or more	1.88	0.00	1.62	2.20
*Race*				
Non-Hispanic White	reference			
Non-Hispanic Black	1.10	0.08	0.99	1.23
Hispanic	1.12	0.05	1.00	1.26
Other	0.90	0.08	0.80	1.01
*Sex*				
Male	reference			
Female	0.91	0.00	0.85	0.97
*Age*				
Age < 56	reference			
Age 56 to 66	0.99	0.73	0.91	1.06
Age 67 +	1.06	0.16	0.98	1.14

*LAR* low anterior resection, *AR* anterior resection, *APR* abdominoperineal resection

Later year of diagnosis (2014–2018 OR 0.85, CI 0.82–0.89; 2018–2021 OR 0.73, CI 0.69–0.76), robotic (OR 0.52, CI 0.50–0.55) and laparoscopic (OR 0.62, CI 0.59–0.65) approach were all associated with lower odds of length of stay greater than 1 week (Table 6).

**Table 6:** Multivariable logistic regression model predicting hospital length of stay of 7 days or greater

	Odds Ratio	p-value	Confidence Interval	
*Year of diagnosis*				
Years 2010–2013	reference			
Years 2014–2017	0.85	0.00	0.82	0.89
Years 2018–2021	0.73	0.00	0.69	0.76
*Operative approach*				
Open	reference			
Robotic	0.52	0.00	0.50	0.55
Robotic, converted to open	1.10	0.17	0.96	1.25
Laparoscopic	0.62	0.00	0.59	0.65
Laparoscopic, converted to open	1.06	0.19	0.97	1.15
*Surgical procedure*				
Partial proctectomy (LAR or AR)	reference			
Coloanal anastomosis	1.04	0.27	0.97	1.12
Total proctectomy (APR)	1.36	0.00	1.30	1.42
Pelvic exenteration	2.46	0.00	2.23	2.72
*Clinical stage*				
Stage 1	reference			
Stage 2	1.29	0.00	1.22	1.36
Stage 3	1.24	0.00	1.18	1.30
*Hospital operative volume*				
Low volume	reference			
Medium volume	0.91	0.00	0.87	0.96
High volume	1.02	0.34	0.98	1.07
*Charlson-Deyo comorbidity score*				
0	reference			
1	1.16	0.00	1.11	1.22
2	1.48	0.00	1.36	1.61
3 or more	1.62	0.00	1.46	1.80
*Race*				
Non-Hispanic White	reference			
Non-Hispanic Black	1.78	0.00	1.67	1.89
Hispanic	1.13	0.00	1.05	1.21
Other	1.02	0.60	0.95	1.09
*Sex*				
Male	reference			
Female	0.72	0.00	0.69	0.74
*Age*				
Age < 56	reference			
Age 56 to 66	1.17	0.00	1.12	1.23
Age 67 +	1.57	0.00	1.50	1.64

*LAR* low anterior resection, *AR* anterior resection, *APR* abdominoperineal resection

Utilizing only minimally invasive approaches, intended laparoscopic surgery had slightly lower odds of readmission (OR 0.91, CI 0.84–0.98), but greater odds of length of stay longer than one week (OR 1.21, CI 1.15–1.28) relative to robotic surgery.

### Mortality and survival

Odds of 30-day mortality were lower in intended robotic and laparoscopic approach compared to open (OR 0.56, CI 0.44–0.72; OR 0.69, CI 0.56–0.85, respectively), as were odds of 90-day mortality (OR 0.63, CI 0.54–0.75; OR 0.72, CI 0.62–0.83, respectively). Year of diagnosis was not a predictor of 30- or 90-day mortality (Table 7).

**Table 7:** Multivariable logistic regression model predicting 30- and 90-day mortality

	30-day mortality				90-day mortality			
	Odds ratio	*p*-value	Confidence interval		Odds ratio	*p*-value	Confidence interval	
*Year of diagnosis*								
Years 2010–2013	reference				reference			
Years 2014–2017	0.94	0.54	0.78	1.14	0.92	0.22	0.80	1.05
Years 2018–2021	0.83	0.09	0.66	1.03	0.90	0.20	0.77	1.05
*Intended operative approach*								
Open	reference				reference			
Robotic	0.61	0.00	0.49	0.77	0.65	0.00	0.56	0.77
Laparoscopic	0.80	0.02	0.66	0.97	0.77	0.00	0.67	0.88
*Surgical procedure*								
Partial proctectomy (LAR or AR)	reference				reference			
Coloanal anastomosis	0.97	0.88	0.70	1.36	1.19	0.14	0.95	1.48
Total proctectomy (APR)	0.95	0.60	0.78	1.15	0.95	0.51	0.83	1.10
Pelvic exenteration	1.10	0.70	0.67	1.81	1.49	0.01	1.09	2.03
*Clinical stage*								
Stage 1	reference				reference			
Stage 2	1.23	0.06	0.99	1.52	1.21	0.02	1.03	1.41
Stage 3	0.95	0.67	0.76	1.19	0.97	0.73	0.83	1.14
*Hospital operative volume*								
Low volume	reference				reference			
Medium volume	0.75	0.00	0.62	0.90	0.79	0.00	0.69	0.90
High volume	0.56	0.00	0.45	0.69	0.66	0.00	0.57	0.76
*Charlson-Deyo comorbidity score*								
0	reference				reference			
1	1.52	0.00	1.25	1.84	1.56	0.00	1.36	1.80
2	2.07	0.00	1.56	2.73	2.36	0.00	1.95	2.86
3 or more	2.70	0.00	1.98	3.69	2.70	0.00	2.15	3.40
*Race*								
Non-Hispanic White	reference				reference			
Non-Hispanic Black	1.44	0.01	1.10	1.89	1.44	0.00	1.19	1.74
Hispanic	1.17	0.37	0.84	1.62	0.93	0.60	0.72	1.21
Other	0.79	0.18	0.56	1.12	0.77	0.04	0.60	0.98
*Sex*								
Male	reference				reference			
Female	0.67	0.00	0.56	0.79	0.73	0.00	0.65	0.83
*Age*								
Age < 56	reference				reference			
Age 56 to 66	2.10	0.00	1.52	2.91	2.32	0.00	1.83	2.94
Age 67 +	6.81	0.00	5.09	9.11	7.50	0.00	6.06	9.29

*LAR* low anterior resection, *AR* anterior resection, *APR* abdominoperineal resection

**Fig. 2:**
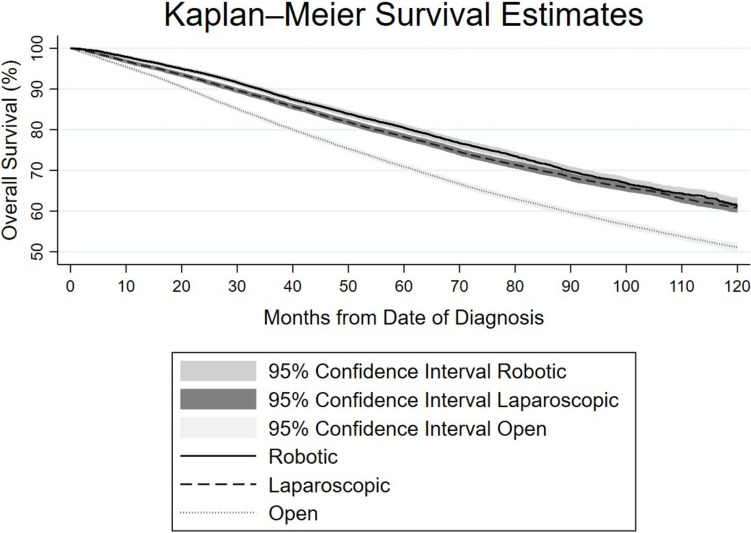
Kaplan–Meier survival curves by surgical approach. Kaplan–Meier survival curves comparing overall survival up 120 months after surgery, stratified by surgical approach: robotic, laparoscopic, and open. Shaded regions represent 95% confidence intervals for each group. Survival differed significantly between approaches (log-rank test, *χ*^*2*^ (2) = 783.98, *p* < 0.0001). Patients who underwent open surgery experienced the lowest survival over time, while those treated with robotic or laparoscopic approaches had significantly better overall survival outcomes

Kaplan–Meier analysis showed that survival outcomes differed significantly by surgical approach (log-rank test χ^2^(2) = 783.98, p < 0.0001). Patients who underwent open surgery had significantly lower overall survival compared to those who underwent laparoscopic or robotic surgery. Both laparoscopic and robotic approaches were associated with improved survival outcomes (Fig. 2). The results of the Cox proportional hazards regression model, which used all-cause mortality as the outcome, indicate that compared to the open operative approach, both intended robotic and laparoscopic approaches were associated with significantly lower hazards of death (HR 0.69, 95% CI 0.66–0.72 for robotic; HR 0.75, 95% CI 0.73–0.77 for laparoscopic). This association remained significant when patients were stratified by cTNM stage I, II, or III. Year of diagnosis was not associated with mortality, as the hazard ratios for the periods 2014–2017 (HR 1.00, 95% CI 0.97–1.03, *p* = 0.88) and 2018–2021 (HR 0.99, 95% CI 0.95–1.04, *p* = 0.79) were not statistically significant compared to the reference period (2010–2013) (Table 8).

**Table 8:** Cox Proportional Hazards Regression Model predicting mortality

	Hazard ratio	*p*-value	Confidence interval		Hazard ratio	*p*-value	Confidence interval		
	All surgical approaches				Minimally invasive only				
***All Patients***									
Intended operative approach									
Open	reference								
Robotic	0.69	0.00	0.66	− 0.72	reference				
Laparoscopic	0.75	0.00	0.73	− 0.77	1.07	0.00		1.03	− 1.12
*Age*									
Age < 56	reference				reference				
Age 56 to 66	1.47	0.00	1.42	− 1.53	1.47	0.00		1.39	− 1.56
Age 67 +	3.08	0.00	2.97	− 3.18	3.38	0.00		3.21	− 3.55
*Year of Diagnosis*									
Years 2010–2013	reference				reference				
Years 2014–2017	1.00	0.88	0.97	− 1.03	1.00	0.88		0.95	− 1.04
Years 2018–2021	0.99	0.79	0.95	− 1.04	1.00	0.97		0.97	− 1.06
***Patients with cTNM Stage I***									
Intended operative approach									
Open	reference								
Robotic	0.69	0.00	0.61	− 0.77	reference				
Laparoscopic	0.72	0.00	0.67	− 0.79	1.05	0.41		0.93	− 1.18
*Age*									
Age < 56	reference				reference				
Age 56 to 66	1.73	0.00	1.52	1.96	1.70	0.00		1.42	2.03
Age 67 +	4.65	0.00	4.17	5.18	5.15	0.00		4.41	6.02
Year of diagnosis									
Years 2010–2013	reference				reference				
Years 2014–2017	0.96	0.39	0.89	1.05	1.00	0.98		0.89	1.13
Years 2018–2021	0.97	0.69	0.86	1.11	0.99	0.95		0.84	1.18
***Patients with cTNM Stage II***									
*Intended operative approach*									
Open	reference								
Robotic	0.77	0.00	0.71	− 0.82	reference				
Laparoscopic	0.83	0.00	0.78	− 0.88	1.07	0.08		0.99	− 1.16
*Age*									
Age < 56	reference				reference				
Age 56 to 66	1.48	0.00	1.37	− 1.59	1.47	0.00		1.31	− 1.65
Age 67 +	3.13	0.00	2.93	− 3.35	3.44	0.00		3.11	− 3.80
*Year of diagnosis*									
Years 2010–2013	reference				reference				
Years 2014–2017	0.99	0.74	0.94	− 1.05	0.96	0.31		0.88	− 1.04
Years 2018–2021	1.00	0.95	0.92	− 1.09	1.01	0.86		0.90	− 1.14
***Patients with cTNM Stage III***									
*Intended operative approach*									
Open	reference								
Robotic	0.70	0.00	0.66	− 0.75	reference				
Laparoscopic	0.74	0.00	0.71	− 0.79	1.05	0.18		0.98	− 1.12
*Age*									
Age < 56	reference				reference				
Age 56 to 66	1.42	0.00	1.34	− 1.50	1.40	0.00		1.28	− 1.52
Age 67 +	2.52	0.00	2.39	− 2.67	2.70	0.00		2.50	− 2.92
*Year of diagnosis*									
Years 2010–2013	reference				reference				
Years 2014–2017	1.00	0.95	0.95	− 1.06	0.99	0.78		0.91	− 1.07
Years 2018–2021	0.99	0.71	0.92	− 1.06	0.98	0.74		0.89	− 1.09

*cTNM* clinical tumor, nodal and metastatic stage, i.e. overall stage

Utilizing only minimally invasive approaches, laparoscopic surgery had greater odds of 30-day (OR 1.35, CI 1.05–1.73) but not 90-day mortality (OR 1.19, CI 1.00–1.42) relative to robotic surgery. Kaplan–Meier analysis showed that survival outcomes differed significantly between robotic and laparoscopic surgery (log-rank test χ^2^(1) = 25.22, *p* < 0.0001), with robotic surgery associated with improved overall survival compared to laparoscopic surgery. On Cox proportional hazards regression modeling, laparoscopic surgery had minimal (but statistically significant) higher hazard of death compared to robotic surgery (HR 1.07, CI 1.03–1.12, *p* = 0.001). However, this association did not remain true when patients were stratified by cTNM stage I, II or III (Table 8).

## Discussion

The use of robotic surgery for rectal cancer resection has increased significantly over the past decade, with the current study demonstrating improved surgical quality over time. This directly addresses the questions regarding the oncologic safety of the robotic approach that emerged after earlier studies. The present data not only corroborates prior findings that robotic surgery is oncologically safe but additionally highlights marked improvements over time. Specifically, we observed a notable increase in the rates of adequate lymph node yield and negative surgical margins, with high-quality resections achieved in 79% of robotic cases in 2019–2021 compared to 69% from 2010 to 2013. This trend persisted even among cases converted to open surgery, with rates improving from 64.7% to 73.2% (p = 0.051). Additionally, the rate of conversions to open surgery declined over the study period, reflecting a nationwide enhancement in proficiency in robotic surgery for rectal cancer. Notably, robotic surgery was associated with significantly improved survival, as evidenced by both lower observed mortality and decreased hazard ratios compared to open and laparoscopic approaches, suggesting that the observed superior surgical outcomes may translate into survival benefits for patients. However, the modest survival advantage initially observed when comparing robotic to laparoscopic approaches in the overall cohort was no longer apparent within stage-specific analyses. This suggests that the survival benefit of minimally invasive surgery over open surgery is robust across stages, but the differential effect between robotic and laparoscopic approaches may be attenuated when controlling for stage.

The Robotic vs Laparoscopic Resection for Rectal Cancer (ROLARR) trial represents the most rigorous randomized controlled data available on robotic surgery in rectal cancer and was conducted between 2011 and 2014 [10]. Despite its strengths regarding rigorous study design, ROLARR likely underestimates the potential advantages of robotic surgery due to its timing, which captured outcomes early in the learning curve of robotic adoption. Participating surgeons were required to have completed only 10 robotic surgeries procedures overall, with the average surgeon in ROLARR having performed roughly 49 robotic rectal cancer cases—standards that may not reflect the expertise now available with greater robotic experience [10, 30]. It demonstrated that robotic surgery was noninferior to laparoscopic surgery in its primary endpoint of conversion to open surgery, with a conversion rate of 8.1% for robotic procedures—nearly identical to the 8.2% conversion rate observed in our study during the same time frame (2010–2013). Secondary outcomes, including postoperative complications, oncological measures, and functional outcomes were similarly comparable between robotic and laparoscopic approaches [10]. However, the cost associated with robotic surgery was significantly higher, which was attributed to longer operative times and robotic instrument expenses [10]. Subsequent analyses of the ROLARR data have identified robotic experience as a significant unaccounted confounder, with learning effects influencing robotic cases more significantly than laparoscopic ones, suggesting surgeons had already saturated their laparoscopic learning curve [29]. Our study suggests that the ROLARR trial’s findings represent a limited snapshot in the evolution of robotic surgery. For example, while the odds of achieving optimal surgical outcomes increased modestly over time for planned open surgery, the planned robotic surgery group demonstrated a far more pronounced improvement. The odds of achieving the composite outcome relative to planned open surgery rose from 1.16 (CI 1.06–1.26) in 2010–2013 to 1.92 (CI 1.81–2.04) in 2018–2021. Furthermore, robotic surgery had superior odds of achieving the composite outcome even when compared only to laparoscopic surgery (2018–2021 OR 1.64, CI 1.49–1.81 vs. OR 1.44, CI 1.30–1.61). This trend, observed even after adjusting for patient, institutional, and procedural factors, underscores the need to reevaluate outcomes as surgical quality can improve over time on a national level. An updated randomized controlled trial reflecting current proficiency would potentially show further reductions in conversion rates and broader improvements in operative and postoperative metrics.

In this context, we must consider the implications of this study at the level of the individual surgeon and institution. As the first documented colorectal robotic surgery was not until 2002, many currently practicing colorectal surgeons did not learn robotics during training, and thus must acquire adequate skills while in practice [31]. There is no standardized process to demonstrate proficiency [32, 33]. Furthermore, the reported number of required proctored cases is exceedingly small, averaging around 3 [32]. For perspective, a graduating general surgery resident must have completed at least 175 simple and complex laparoscopic cases under the guidance of an attending physician [34]. Once credentialed, ongoing appraisal is similarly sparse and varies from hospital to hospital, with roughly 7 cases required annually to maintain robotic access and continuous outcome monitoring inconsistently implemented [32]. Although robotic credentialing requirements are varied and often minimal, the advanced data capture capabilities of the robotic system itself—including video review, surgical tool motion, and precise operative times—provides an opportunity for unparalleled review of technical performance and proficiency [35, 36].

In addition to standardized training, field-specific guidelines are also necessary to contextualize the unique demands of a given surgical pathology [37–39]. The European Society of Coloproctology has recently released guidelines specifically on robotic training in colorectal surgery [40], but other societies such as American Society of Colon and Rectal Surgeons has provided only limited commentary in their consensus guidelines without practical insights into surgical technique [28]. The Accreditation Council for Graduate Medical Education has set no formal robotics requirements for general surgery residents or colorectal fellows and currently robotic case numbers are captured only as a qualifier added to traditional laparoscopic case logs [34, 41]. However, some robust efforts at graduate teaching have been made: for example, the Association of Program Directors for Colon and Rectal Surgery paired with Intuitive Surgical have devised a robust annual training for colorectal fellows with various robotic experience [42]. Since the use of robotics is increasing, it would be valuable for credentialing bodies to implement evidence-based standards for its inclusion in curricula in the form of minimum case requirements to assure that training programs provide the necessary educational exposure to meet the needs of future colorectal surgeons.

While robotic surgery may offer superior oncologic outcomes in rectal cancer when performed by experienced surgeons, its high cost raises important questions about equitable access. Prior studies have highlighted disparities in access to robotic procedures across various populations [43–46]. For example, Black, Hispanic, and Medicaid-insured patients were less likely to undergo radical prostatectomy at hospitals offering robotic surgery [43]. Similarly, an NCDB analysis for robotic colon cancer surgery found that patients with private insurance had higher odds of receiving robotic surgery, whereas older and Black patients were less likely to undergo these procedures [44]. Another NCDB study examining colon and rectal cancer surgeries revealed that patients with Medicaid or those who were uninsured had significantly lower odds of receiving robotic operations compared to privately insured patients [45]. These findings, along with our data, underscore the need to address systemic inequities to ensure that advancements in robotic surgery benefit all patients, irrespective of age, race, or socioeconomic status so that we do not further widen gaps in access to the highest quality care and outcomes that already exist.

The current study is limited in its use of a composite outcome as a proxy for high-quality oncologic resection, as it does not include all other known prognostic factors, such as total mesorectal excision grade or pathologic factors like lymphovascular invasion or perineural invasion. However, the prognostic implications of adequate lymph node yield and surgical margins are well established [23–27], and are standard metrics used by colorectal surgeons and pathologists to characterize the success of an oncologic resection [28]. Furthermore, given the limitations inherent to the data available in the NCDB, we are unable to comment on other important factors commonly considered in the discussion of robotic surgery, such as operative time or surgical cost. We would argue, given the superior oncologic surgical resections compared to laparoscopic and open approaches, these factors are less critical but would still provide helpful context from a healthcare resources perspective. Another limitation is the inherent preoperative selection bias in choosing a surgical approach. Factors such as prior abdominal surgeries, concerns regarding tolerance of pneumoperitoneum, or other clinical judgments may predispose certain patients—particularly those who are less fit or have more complex anatomy—to undergo open surgery. This bias may be more pronounced in later years as minimally invasive surgery gained broader acceptance. Unfortunately, these clinical considerations are not captured in the NCDB and could not be accounted for in our analysis. Furthermore, the NCDB groups together confirmed open surgeries and cases with an unspecified surgical approach under a single category, which introduces the possibility that some cases classified as open may, in fact, have been minimally invasive procedures misclassified due to coding limitations.

This study challenges the notion that robotic surgery is merely non-inferior to traditional laparoscopic or open techniques for rectal cancer. Instead, it demonstrates that, with adequate clinical expertise, the unique technical advantages of robotic surgery translate into significantly improved oncologic outcomes. However, realizing these benefits will require our field to address critical gaps in training and access: our findings demonstrate that the robot alone cannot compensate for operator or institutional inexperience, as improved outcomes over time highlight the essential role of experience in maximizing the benefits of robotic surgery. There is space for training programs to ensure a standardized approach to proficiency and for accrediting bodies to ensure adequate cases to provide opportunities to achieve proficiency. Simultaneously, institutions can prioritize expanding robotic access and investing in comprehensive training programs for current staff to deliver optimal care. Without these measures, the promise of robotic surgery to improve outcomes and ensure equitable care in rectal cancer may remain unrealized. The prior decade has cemented the adoption of robotic surgery in rectal cancer, despite the paucity of compelling data for its support early on, necessitating a hasty retrospective alignment of institutional priorities, training structures, and resource allocation to meet the current landscape.
